# Comparative analysis of bone healing in subcritical defects with air turbine and electric handpiece in a rat model

**DOI:** 10.1371/journal.pone.0312280

**Published:** 2024-12-30

**Authors:** Izabella Sol, Henrique Hadad, Tatsuya Henrique Kano, Karen Rawen Tonini, Martina Andreia Lage Nunes, Daniela Ponzoni

**Affiliations:** Department of Diagnosis and Surgery, São Paulo State University (UNESP), School of Dentistry, Araçatuba, São Paulo, Brazil; Universidade de Trás-os-Montes e Alto Douro: Universidade de Tras-os-Montes e Alto Douro, PORTUGAL

## Abstract

Rotatory devices are essential in clinical surgical practice, however, depending on the different systems available, their function can impact bone repair and postoperative responses on varying scales. This impact underscores the need to explore new techniques aiming to enhance bone repair. This study aimed to assess the immediate and delayed effects on bone healing in subcritical bone defects using both air turbine and an electric handpiece. For this purpose, 40 male *Wistar* rats were allocated into two groups. The Control Group (CG) had bone defect made using an air turbine device, while the Experimental Group (EG) had defects made using an electric handpiece. Ten animals were sacrificed for each time of evaluation. Bone neoformation, microstructure, and collagen organization were assessed ate 7, 15 and 30 days postoperative. Inflammatory profiling was conducted at 7 and 15 days. Immediate thermal osteonecrosis were evaluated after the use of rotary systems. Multivariate analysis was used to access statistical differences. The EG exhibited enhanced parameters of bone neoformation in all analyses, with statistical difference between 15 and 30 days (P = .0002) and in comparison with CG in 30 days (P = .0009). A reduced number of inflammatory cells and increased angiogenesis in the initial periods was seen in EG, corroborating the consistent values of collagen type 1 and a decrease of collagen type 3 over times. Immediate thermal osteonecrosis was statistically higher for the CG (P < .05), which showed adequate neoformation of subcritical defects but consistently lower values than those found in the EG. These data suggest that the electric handpiece demonstrated more bone repair area, proving to be an excellent alternative to surgical practice.

## Introduction

Enhancing bone repair processes, whether under local or systemic conditions, is an ongoing and essential pursuit. The aim is to offer alternatives that improve the precision and safety of oral surgeries. Various experimental models are employed to explore the diverse mechanisms of bone healing [1,2].

Bone repair involves a series of processes that typically restore the injured bone to its original cellular structure, thereby recovering its initial biomechanical properties. However, this restorative process may be negatively influenced by physiological, environmental, or local factors [3]. Ostectomy represents a critical step in oral surgery. The use of rotatory instruments can induce local trauma, potentially leading to delays in the repair process [4,5].

It is believed that osteonecrosis generated during cutting procedures is dependent both temperature and time [6]. Other factors influencing heat production include the pressure applied during drilling, drill characteristics, drilling dynamics (continuous or intermittent, depth), the cooling technique used, the speed of the rotating instrument, magnitude and duration of the temperature reached, and bone density [7–12]. Due to the anisotropic nature of bone [6,9], a local increase in temperature can lead to bone necrosis. This leads to denaturation of enzymatic and membrane proteins, degeneration of osteocytes, decreased osteoclastic and osteoblastic activity [8,9,13], and limits blood flow [2,6]. The threshold temperature for causing these damages is not yet clear, but it is accepted that a temperature of 47°C sustained for 1 minute, even with correct irrigation, can lead to bone damage [9,14–16]. It has become more clear that choosing the correct rotary instrument in oral surgery, especially when greater surgical difficulty is expected, is essential.

Various rotatory systems are available in the market, and research on the influence of their different rotations on bone repair is well-documented in the literature [17–19]. The piezoelectric system has demonstrated to be a good option in pre-clinical [2,7,12] and clinical studies [5] However, this equipment is costly, necessitates training, and extends the duration of the surgery [5,13,20]. The conventional turbine (air turbine) consistently shows worse results when compared to other systems with lower rotation [5,21,22]. The electric handpiece is relatively unexplored in the field of oral surgery, but its higher cutting efficiency has already been proven in other fields [4,13,23].

Considering that post-surgical sequelae represent a burden on patients and on the healthcare system [24], it is necessary to search for new treatments that can help overcome complications or accelerate bone regeneration. The primary aim of this study is to comparatively evaluate bone neoformation in subcritical bone defects using an air turbine and an electric handpiece through histometric and bone microstructure evaluations. The second aim is to compare the differences in the immediately thermal osteonecrosis caused by the ostectomy and the inflammatory response between the two rotatory systems.

## Material and methods

### Animals and ethical approval

This study was carried out in strict accordance with the recommendations of Ethical Principles of Animal Experimentation (Protocol CEUA-FOA #0516/2021) and followed normative regulations of the National Council for the Control of Animal Experimentation (CONCEA) in Brazil [25], and the ARRIVE Guidelines [26]. The animals were maintained in appropriate open cages at the Central Animal Facility of São Paulo State University, School of Dentistry, Araçatuba, São Paulo, Brazil, with a maximum of 4 rats in each one, under controlled temperature (22 ± 2°C) and light cycle (12 h dark/light) room, with no restriction of water and food intake.

### Experimental groups

Based on previous findings of bone neoformation [27], the power test was conducted using the website *http*:*//www*.*biomath*.*info/power/prt*.*htm* to determine the sample size. With a standard deviation of 16.7 at a 5% significance level, the test achieved an 80% power in a one-tailed hypothesis test. The sample size was determined to be n = 10. Forty male rats (*Wistar*) tibias were randomly categorized into two groups based on the handpiece employed: CG (control group–air turbine); EG (experimental group—electric handpiece).

Each group were evaluated in 4 times, as follows: immediately after surgical procedure, 7, 15 and 30 days after procedure. All animals received both treatments in one tibia chosen by randomization, totalizing the euthanasia of 10 animals per time.

### Surgical procedure: Subcritical bone defect

**Fig 1:**
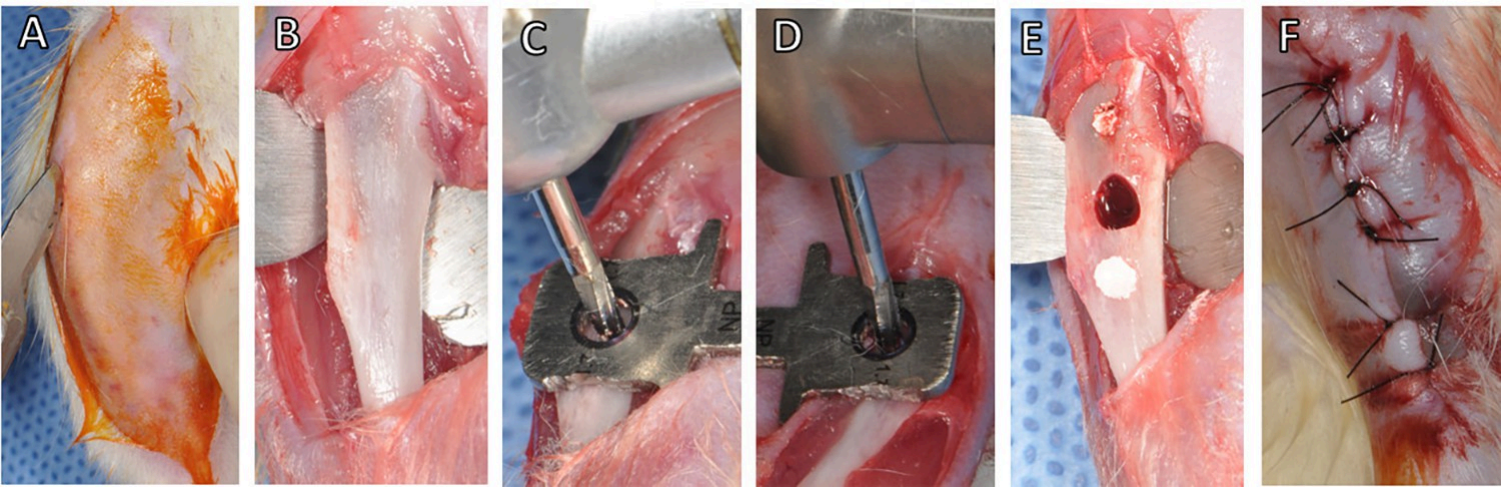
Experimental surgical procedure. Linear incision in the medial region of the tibia (A). Exposure of the proximal anteromedial metaphysis of the tibia (B). Central cavity perforation with an air turbine (C) and with an electric handpiece (D). Final representation of the sites containing a central defect and two lateral defects filled with gutta-percha (E). Suture (F).

Two calibrated surgeons (DP and IS) performed all the surgical procedures. The animals were under appropriate fasting for 12 hours. After individual weighing, the rats were anesthetized with IM injection of 1% ketamine chloride (80mg/kg) (Dopalen, Agribrans do Brasil LTDA, SP, Brazil) in association with 2% xylazine chloride (15mg/Kg) (Anasedan, Agribrans do Brasil LTDA, SP, Brazil). After trichotomy and antisepsis with 1% polyvinylpyrrolidone of the region of medial tibiae, the animals were placed on the surgical table by supine position (Fig 1A). Local anesthesia was made to improve pain and bleeding management (Mepiadre 2%, DFL, Rio de Janeiro, RJ, Brazil). A 2cm linear incision by layers was performed, exposing the anteromedial metaphysis of both tibias (Fig 1B).

A surgical perforation guide (Kit Surgical Guide Signo Vinces, Campo Largo, PR, Brazil) was used to standardize the distances between the subcritical bone defects: the main defect was made with a dental bur (HP 702 tungsten carbide, Kavo, Joinville, SC, Brazil) coupled with the air turbine (CG; Kavo 505c, Joinville, SC, Brazil at 450.000rpm) or the electric handpiece (EG; NSK, Bauru, SP, Brazil at 200.000rpm), introducing only the active portion of the bur to pass through the superficial cortical of the tibia; two peripheric defects were filled with gutta-percha only to facilitates the localization of bone defects during analysis (Fig 1C–1E). A new bur was used for each group and period, with external irrigation using sterile saline solution. The incision was closed by layers using 4.0 absorbable suture (Vycril 4.0, Ethicon, Johnson Prod., São José dos Campos, SP, Brazil) and 5.0 nylon suture (Mononylon, Ethicon, Johnson Prod., São José dos Campos, SP, Brazil) (Fig 1F). At immediately postoperatively, each animal received a single intraperitoneal dose of 0.2 mg/mLpenicillin G benzathine (Pentabiótico Veterinário Porte, Fort Dodge Saúde Animal Ltda., Campinas, SP, Brazil).

### Histological analysis

Euthanasia was performed at 0, 7, 15, and 30 days using an overdose of anesthetic (10 animals per group and time). All collected samples were fixed in 4% paraformaldehyde for 48h to ensure tissue preservation. Two representative specimens were isolated before demineralization, then rinsed for 24 hours in running water, and stored in 70% alcohol for micro-computed tomography (μCT) analysis. Subsequently, all the samples were washed in water, followed by 8-weeks of decalcification in 10% ethylenediaminetetraacetic acid (EDTA). After a 12-hour washing period, the samples were dehydrated through a series of alcohol concentrations (70% up to 100%), diaphanized in xylene, and embedded in paraffin. In the sequence, microtome cutting at 5μm was performed on the samples. Histological sections were then mounted and stained with Hematoxylin and Eosin (HE) for the analysis of bone neoformation and inflammatory response. Evaluation of extracellular collagen deposition was conducted using the Picro Sirius Red (PSR) stain.

### Histometric analysis

An individual researcher, who was both calibrated and blinded to the treatments, conducted the analysis. Qualitative assessment involved the examination of medullary and cortical areas, as well as the identification of connective tissue. For quantitative analysis, images were acquired at 25x magnification (Opton 3984) and analyzed using ImageJ software (National Institutes of Health, Bethesda, MD, USA) [12]. Utilizing the polygon tool, the total area was measured (μm^2^), followed by the quantification of new bone formation and presence of connective tissue.

The bone microstructure was assessed using micro-computed tomography (μCT) (Skyscan 1174, Bruker, Kontich, Belgium). During scanning, the following parameters were applied: 6μm pixel size, 70 kVp, 0,5mm aluminum filter, and 0.5° rotation step. After image processing, only the subcritical bone defect was analyzed. It were measured the morphometric parameters: Bone volume (BV); Percent bone volume (BV/TV); Trabecular thickness (Tb.Th), number (Tb.N) and separation (Tb.Sp); and Total porosity (Po.tot) [28]. The collected data were tabulated and subjected to analysis.

The degree of immediate thermal osteonecrosis caused by the dental bur in the subcritical bone defect in each group was quantified in the animals euthanized immediately following the surgical procedure. ImageJ software was employed to measure the upper, middle, and lower thirds of the defect, and the averages were calculated. The margins of osteotomy were assessed and categorized as smooth, mildly irregular, or irregular [13].

To quantify the amount of collagen deposition and expression, the slices with PSR stain passes through interpolarization 10x magnification (Leica DM 4000B). One slice per animal was select. Using the software LAS V4 12 (Leica Application Suite V4), the total area and percentage of collagen type I (Col-I) and type III (Col-III) were calculated in CG and EG at 7, 15 and 30 days.

Inflammatory cell (lymphocyte) and vessel counts were conducted on samples from the 7th and 15th periods. Two slices per animal were chosen to assess three regions of the defects (central region, right side, and left side) under a 100x magnification (DM 4000 B, Leica, Mannheim, Germany). Photomicrographs were analyzed using ImageJ software, with a 130-point grid, considering only cells that intersected the line [29].

### Statistical analysis

Data were analyzed using Prism software (GraphPad, La Jolla, CA, USA). After confirmation of normal distribution (Shapiro-Wilk, P>.05), two-way analysis of variance (ANOVA) with *post hoc* Tukey’s tests were applied to test the data of histometric, μCT and collagen deposition. Paired t-Test were applied for inflammatory response comparison. One-way analysis of variance (ANOVA) with *post hoc* Tukey’s tests was used to analyze the immediately necrosed area. A P-value less than 0.05 (P < .05) was considered statistically significant in all analysis.

## Results

### Bone microstructure shown faster bone healing with electric handpiece

**Fig 2:**
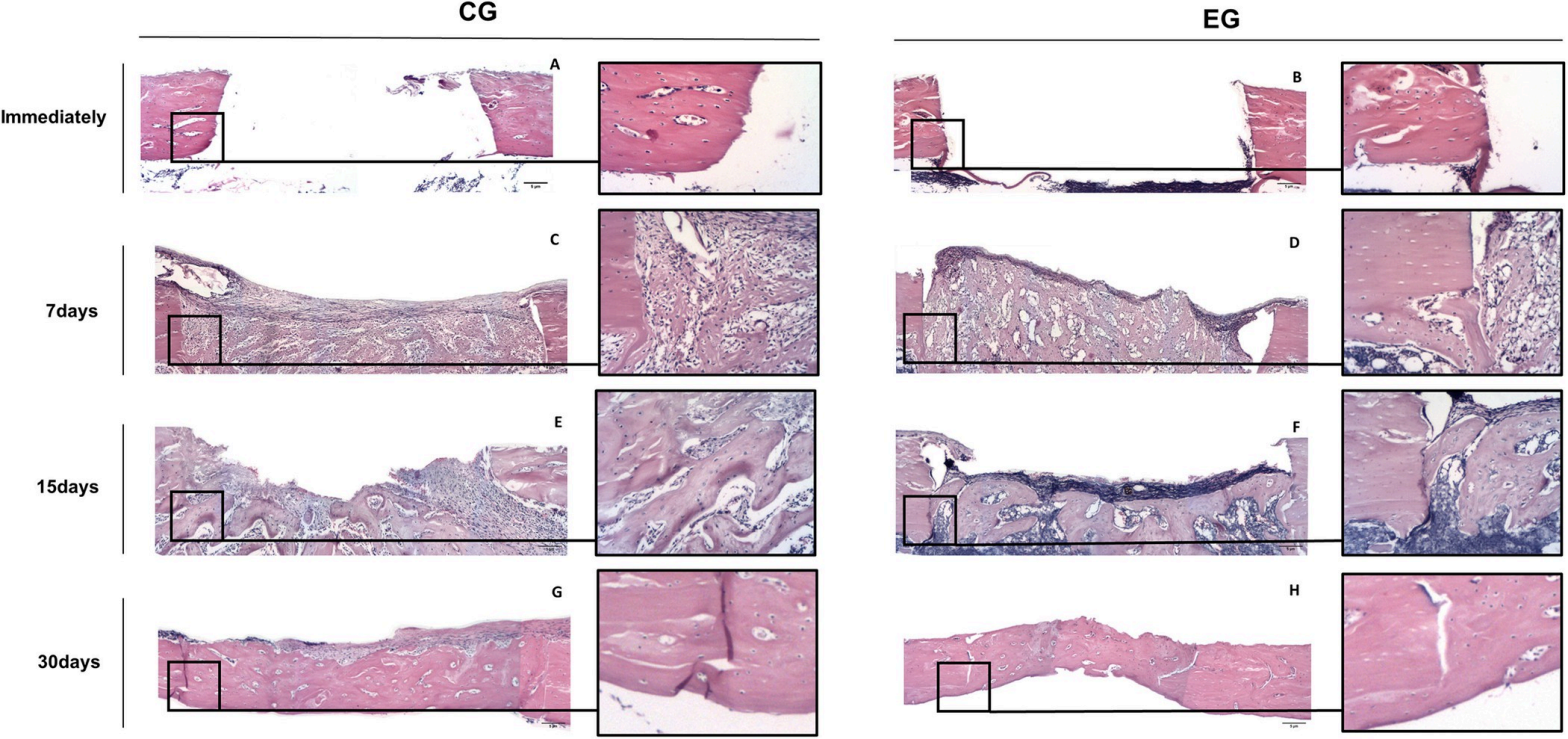
Panoramic reconstructions of photomicrographs of bone neoformation. Repair process in the CG (left) and EG (right) groups at immediate (Im.) time (A,B), 7 (C,D), 15 (E,F) and 30 (G,H) days. Distinct patters in bone healing are observable. The EG exhibits accelerated proliferation of woven bone and faster differentiation at 7 and 15 days. At 30 days, increased amount of connective tissue is present in CG. The trabecular bone patterns are highlighted in the boxes adjacent of the panoramic reconstructions. HE staining with a magnification of 25x and a scale of 5μm.

**Fig 3:**
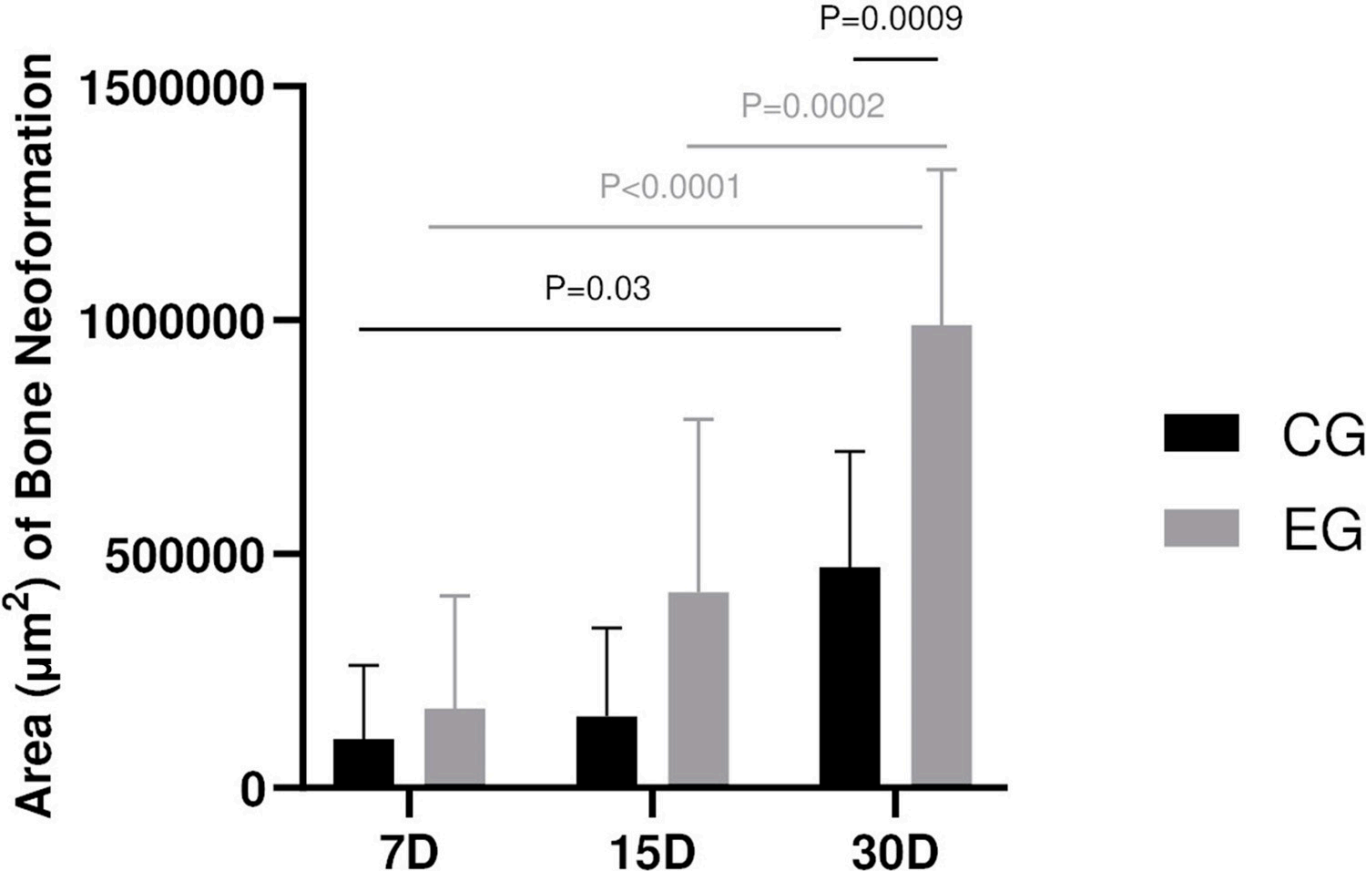
Comparative graph of histometric analysis. Means and standard deviation of the newly formed bone at 7, 15 and 30 days in the CG and EG groups. Statistical differences (P < .05) are highlighted at CG 7 and 30 days, EG 7 and 30 days, EG 15 and 30 days, and at 30 days between CG and EG.

A complete bone repair was evident in all subcritical bone defects. However, qualitative analysis revealed a reduced presence of connective tissue and more bone neoformation in the EG, indicating accelerated bone healing (Fig 2A–2H). The thermal osteonecrosis was significantly higher in CG (P < .05), with smoother margins observed in the majority of the slices (Fig 2A and 2B). Histometric assessment of bone neoformation showed noteworthy results. The EG exhibited higher area of new bone formation in all time points, with a significant difference noted at 30 days compared to the CG (P < .001) (Figs 3 and S1).

**Fig 4:**
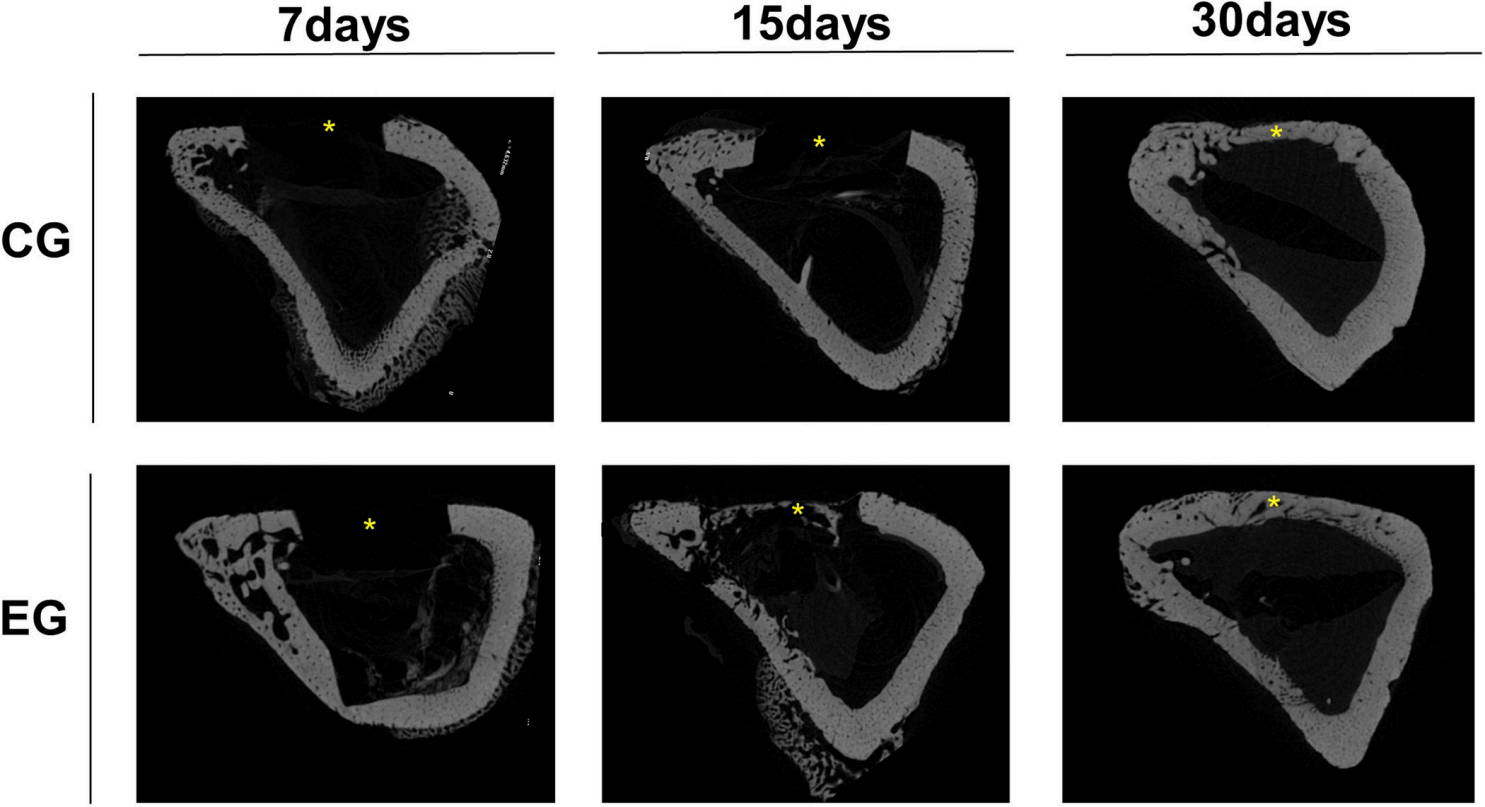
μCT scanning in three-dimensional sagittal view images. Representative photomicrographs at 7, 15 and 30 days of the CG and EG groups. The asterisks (*) represent the defect analyzed. EG revealed enhanced formation and bone differentiation.

**Fig 5:**
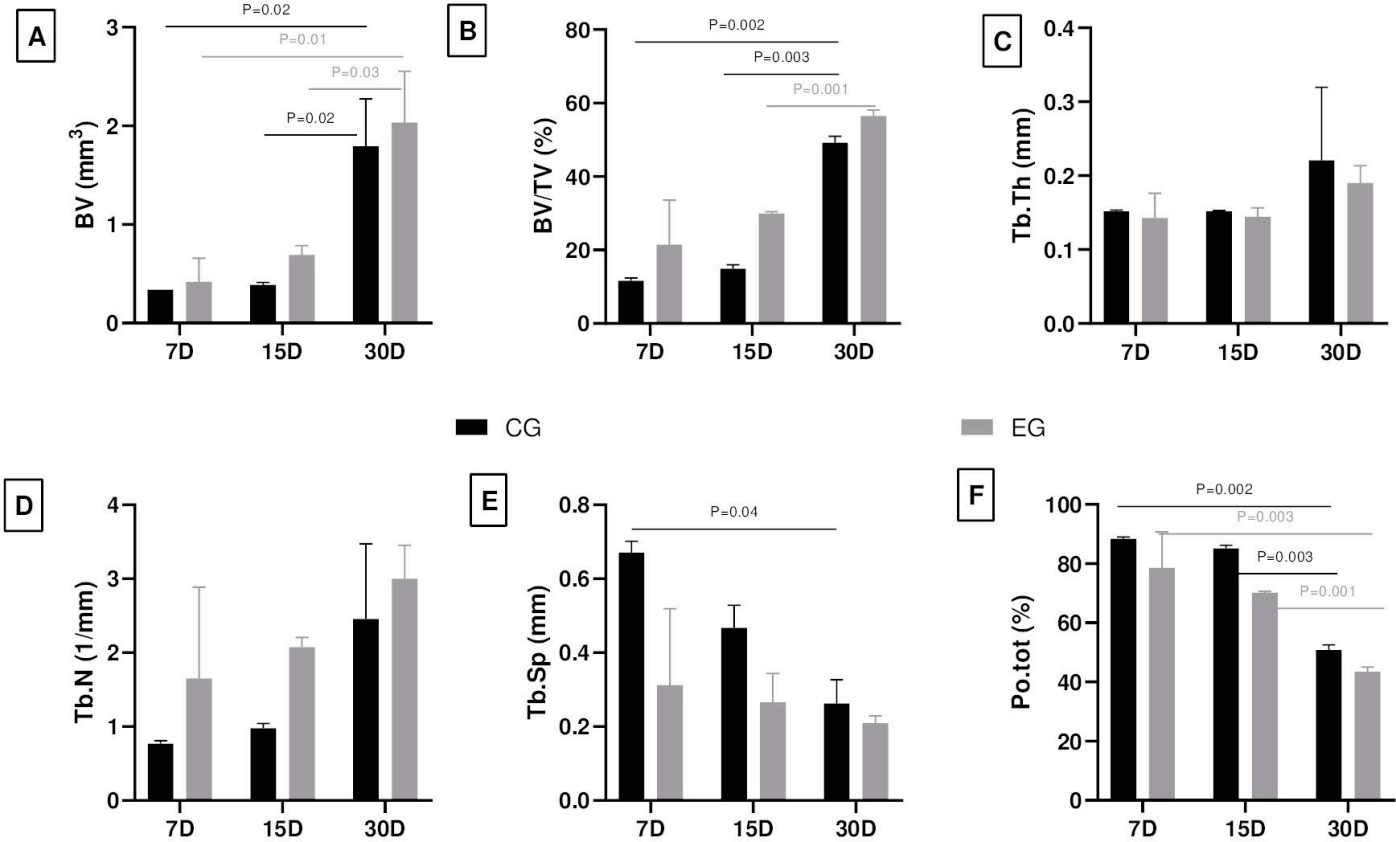
Graphs of bone microstructure parameters. Means and standard deviation at 7, 15 and 30 days in the CG and EG groups. Bone volume–BV (A), Percent bone volume–BV/TV (B), Trabecular thickness–Tb.Th (C), Trabecular number–Tb.N (D), Trabecular separation–Tb,Sp (E), and Total porosity–Po.tot (F). Intragroup statistical differences (P < .05) are highlighted in each graph.

The μCT analysis of bone microstructure revealed complete repair in both groups (Fig 4). Parameters associated with bone neoformation indicated a progressive bone healing in both CG and EG (Figs 5 and S1). Although no significantly differences were observed between the groups, the EG consistently exhibited higher values in all parameters of bone neoformation in the analyses.

### Air turbine and electric handpiece generate different inflammatory responses and collagen expression

**Fig 6:**
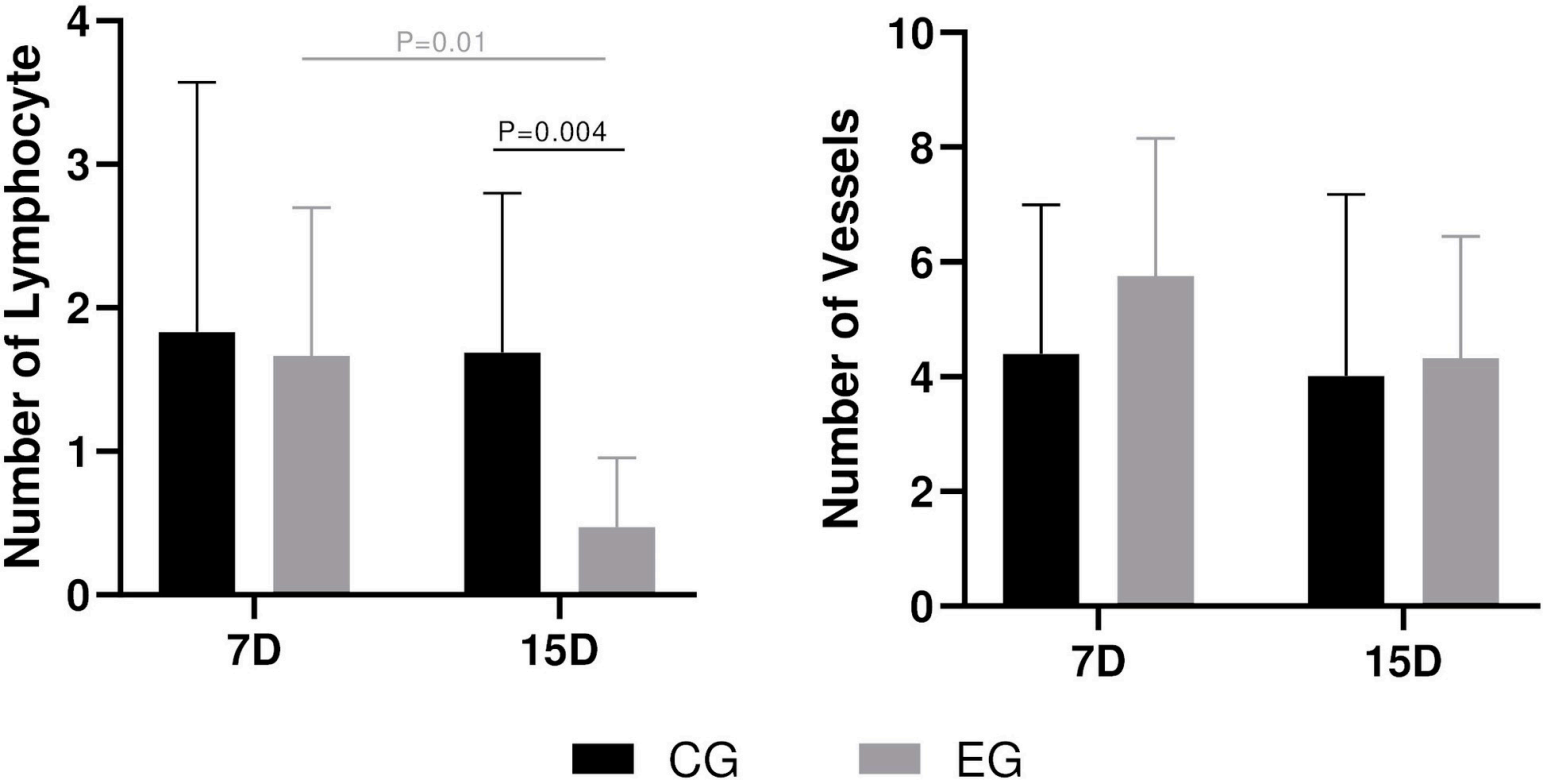
Graphs of inflammatory response. Means and standard deviation of 7 and 15 days in the CG and EG groups. Statistical differences (P < .05) are highlighted in each graph.

**Fig 7:**
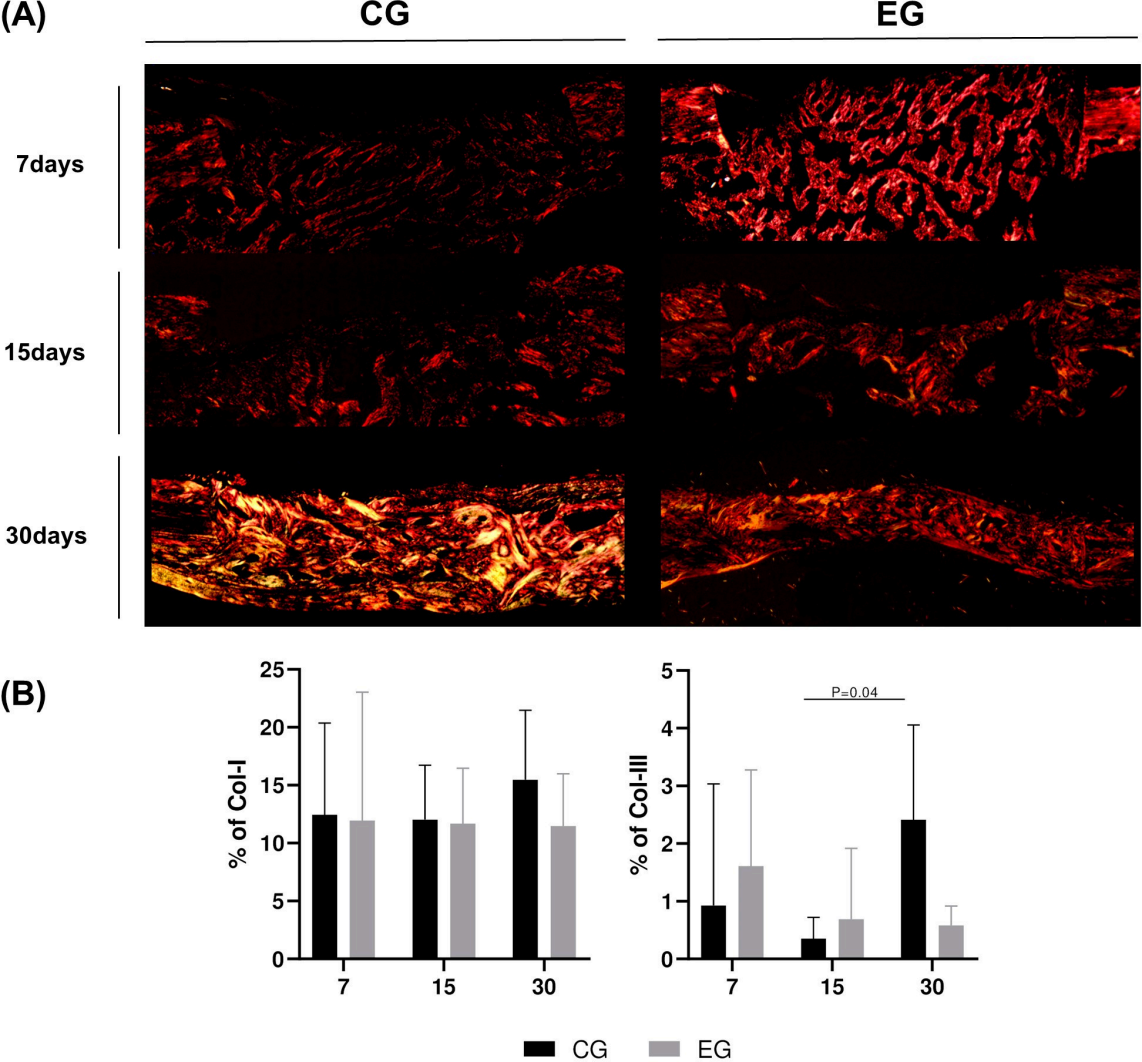
Picro-Sirius red staining and collagen fibers analysis. (A) Representative microphotography at 7, 15 and 30 days of the CG and EG groups. PSR staining with 10x magnification. The collagen fiber polarization illustrates more intense quantity of maturate type I collagen fibers (red) in EG group at all times analyzed. (B) Semi-quantification of Col-I (red) and Col-III (green) fibers in CG and EG groups.

Evident differences were apparent in both groups. The CG exhibited higher lymphocyte counts in both analyzed periods (Table 1, Figs 6 and S1) with statistical differences at 15 days analysis with EG (P = .004). The number of vessels was higher in the EG, but not statistically different. These observations were further supported by the PSR stain. CG showed reduced Col-I at 7 and 14 days, while the EG demonstrated consistent values. The expression of Col-III, indicating early phase in bone neoformation, increased over time in CG and decrease in EG (Figs 7 and S1). Multivariate analysis revealed no statistical differences intragroup in collagen expression analysis.

**Table 1:** Inflammatory response. Means and standard deviation of the inflammatory response (number of lymphocytes and vessels) in CG and EG groups.

	Lymphocyte (n)			Vessel (n)		
	CG	EG	P-value	CG	EG	P-value
7 days	2.6 ±2.3	2.3 ±1.6	P = .77	4.8 ±2.0	6.3 ±1.2	P = .23
15 days	1.7 ±1.0	0.7 ±0.4	P = .004	4.4 ±2.8	4.8 ±2	P = .76
P-value	P = .26	P = .01		P = .72	P = .17	

CG–control group; EG–experimental group; n–number.

## Discussion

The electric handpiece exhibited a lower inflammatory response and faster bone neoformation, although complete bone healing was achieved in both experimental groups. Animal models offer a closer evaluation of the dynamics of bone healing events, with complete repair in 21 days [27,30]. The incorporation of monocortical defects in these models allows simulation of spontaneous healing similar to alveolar repair [24,27,31,32]. The choice of the rat model with tibia subcritical defect provides a safe and uneventful repair, where only the inflammatory response generated during the surgical procedure influences the bone healing process.[27] Limitations such as size of the defect, need for microscopic evaluation and shorten healing are reported with these model studies [30]. Clinical studies to corroborate the findings described in this research are needed. We emphasize that this study is a pioneering contribution to the field, with no other study in the literature comparing the repair of subcritical bone defects between air turbine and electric handpiece.

Comprehending the characteristics of bone healing is imperative to guide therapeutic protocols. Various factors, such as heat generation, have the potential to modify the repair process. The osteotomy procedure significantly influences postoperative outcomes, with surgical trauma posing risks of pseudoarthrosis, infection, and delayed repair [7]. Our findings underscore the impact of the handpiece choice on the healing process, revealing a larger thermal osteonecrosis following the use of the air turbine. This outcome translated to lower rates of bone neoformation, as evidenced by both histometric and μCT analyses.

The air turbine is characterized by a rotational speed ranging from 300.000 to 450.000 rotations per minute (rpm) during use, accompanied by vibration, noise, and low torque [23,33]. Despite these drawbacks, air turbines remain prevalent in dental procedures [13]. On the other hand, the electric handpiece has high torque, minimized vibration and noise, with rotations reaching up to 200.00 rpm [13,33]. The fields of prosthetics and endodontics have witnessed greater cutting efficiency and reduced heat production with the electric handpiece [22,23,33–35]. However, its application in maxillofacial surgery remains relatively unexplored. Notably, the measurement of heat generated during osteotomies, as suggested in previous research [6,36], was not conducted in this study. We propose incorporating this analysis in future investigations to precisely correlate this factor with the bone healing. Furthermore, both groups successfully completed the repair within the times evaluated, reinforcing our hypothesis of temperature maintenance below the critical harming value for bone by using correct refrigeration and new instruments during the experiment [6,9,36].

The utilization of piezoelectric devices has demonstrated a positive correlation with enhanced bone healing, attributed to lower heat production and optimized cutting efficiency compared to air turbines [2,5,22,23]. Despite this, no significant difference in alveolar repair time is observed between these systems [12,27]. While acknowledging methodological variations among studies, including those different from the approach adopted in this work, a clear divergence in reported results is evident in the literature. The comparison of drilling osteotomy with piezosurgery is well stablished in the oral surgery literature. The bone healing dynamics are similar between the two systems, with no differences in bone remodeling markers and the percentage of bone neoformation [2,12,27]. Due to these consistent findings, we did not include a piezoelectric comparison group, focusing our study on a rotary system with greater cutting efficiency that is still underexplored in the field. Generally, instruments with superior cutting efficiency exhibit greater precision and generate less heat [23].

In accordance with existing literature, the electric handpiece showed a visible advancement in the repair of subcritical bone defects. Histological evaluation, recognized as the gold standard for *in situ* cell analysis [7], enabled the observation of reduced inflammatory response and higher angiogenesis in the EG (Figs 2 and 6, Table 1). Additionally, notable differences in the amount of bone neoformation became apparent, implying that the surgical trauma associated with the two distinct systems influenced the bone response. These findings were further supported by a statistically significant increase in immediate thermal osteonecrosis observed in the CG. We hypothesize that the cumulative impact of these factors plays a pivotal role in the local postoperative response. These insights must be considered carefully and analyzed in future randomized clinical trials (RCTs), designed to assess and validate the clinical efficacy of electric handpiece in oral surgery. Based on the presented data, we anticipate a reduced inflammatory phase and improved postoperative recovery. A recent RCT demonstrated enhanced clinical outcomes with the electric handpiece compared to air turbine in third molas extractions [37]. The alveolar repair with the different rotatory systems showed no statistical differences, notable reduction in the inflammation parameters were observed [37].

Micro-tomography evaluation stands as a standardized method to quantifying the three-dimensional bone microstructure [12]. Despite the absence of a statistical difference between groups, the graphical results clearly illustrate more repair area in EG. The observed decrease in porosity and increase in bone volume within this group indicate a higher rate of local healing. Moreover, parameters noted in the EG, such as smaller trabecular spacing and thicker trabeculae, are indicative of greater microhardness and improved bone quality. Corroborating these analyses, the consistent Col-I type (product of osteoblastic bone formation) values reinforced the enhanced bone healing in the EG. The expression of Col-III type (present in connective tissues) in the bone indicates the early stages of bone formation, linked to osteoblast recruitment and early differentiation [38]. This indicates a slower repair process observed in the CG, contributed by the lower percentage of bone neoformation in the histometric analysis. These results should be interpreted with caution, but they clearly suggest that different repair processes are expected when using the rotary systems analyzed in this research.

Several studies emphasize the significance of micromechanical investigations, including compression, tension, and microhardness tests, to comprehensively assess bone properties [1,7,24]. Shear tests provide a quantitative means to evaluate the bone regeneration process over time. In the context of monocortical defects, the bone repair process can be identified and quantified up to the third week, with no statistical differences observed thereafter. This indicates that, once the defect is populated with bone trabeculae, there is minimal or no alteration in their generation, and the newly formed tissue attains shear strength comparable to the original state [24]. Although this analysis is not present in our study, the process described aligns with our results. The presence of immature bone at 15 days evolved into complete bone remodeling by 30 days. Furthermore, the selected evaluation times in this study are justified.

Considering the unique outcomes derived from this study, we advocate for the implementation of randomized clinical trials in surgical procedures involving osteotomies, such as the extraction of impacted third molars, to observe the clinical effects that may or may not be associated with our findings.

## Conclusion

In this experimental model, the electric handpiece demonstrated enhanced efficiency in bone healing, evidenced by accelerated bone neoformation, reduced inflammatory response, and enhanced angiogenesis during the initial stages of repair. Additionally, the optimization of bone microstructure parameters further supports the advantages of the electric handpiece over the air turbine.

## Supporting information

S1 Fig Excel spreadsheet with numerical data for Figs 3 and 5–7. (XLSX)
